# Cost-effectiveness analysis of a two-way texting (2wT) intervention to improve ART retention among newly-initiated antiretroviral therapy clients in Malawi

**DOI:** 10.1093/oodh/oqae030

**Published:** 2024-12-02

**Authors:** Christine Kiruthu-Kamamia, Hiwot Weldemariam, Mirriam Chipanda, Jacqueline Huwa, Johnnie Seyani, Harrison Chirwa, Aubrey Kudzala, Agnes Thawani, Joseph Chintedza, Odala Sande, Geldert Chiwaya, Hannock Tweya, Milena Pavlova, Wim Groot, Caryl Feldacker

**Affiliations:** Maastricht Economic and Social Research Institute on Innovation and Technology, United Nations University, Boschstraat 24, 6211 AX, Maastricht, Netherlands; Lighthouse Trust, Kamuzu Central Hospital Area 33 Mzimba Street. P.O. Box 106, Lilongwe, Malawi; International Training and Education Center for Health, 325 9th Avenue, Seattle, Washington 98104, USA; Department of Epidemiology, University of Washington, 3980 15th Ave NE, Seattle, Washington 98195, USA; Lighthouse Trust, Kamuzu Central Hospital Area 33 Mzimba Street. P.O. Box 106, Lilongwe, Malawi; Lighthouse Trust, Kamuzu Central Hospital Area 33 Mzimba Street. P.O. Box 106, Lilongwe, Malawi; Lighthouse Trust, Kamuzu Central Hospital Area 33 Mzimba Street. P.O. Box 106, Lilongwe, Malawi; Lighthouse Trust, Kamuzu Central Hospital Area 33 Mzimba Street. P.O. Box 106, Lilongwe, Malawi; Lighthouse Trust, Kamuzu Central Hospital Area 33 Mzimba Street. P.O. Box 106, Lilongwe, Malawi; Lighthouse Trust, Kamuzu Central Hospital Area 33 Mzimba Street. P.O. Box 106, Lilongwe, Malawi; Lighthouse Trust, Kamuzu Central Hospital Area 33 Mzimba Street. P.O. Box 106, Lilongwe, Malawi; Lighthouse Trust, Kamuzu Central Hospital Area 33 Mzimba Street. P.O. Box 106, Lilongwe, Malawi; Lighthouse Trust, Kamuzu Central Hospital Area 33 Mzimba Street. P.O. Box 106, Lilongwe, Malawi; International Training and Education Center for Health, 325 9th Avenue, Seattle, Washington 98104, USA; Department of Global Health, University of Washington, 3980 15th Ave NE, Seattle, Washington 98105, USA; Maastricht Economic and Social Research Institute on Innovation and Technology, United Nations University, Boschstraat 24, 6211 AX, Maastricht, Netherlands; Maastricht Economic and Social Research Institute on Innovation and Technology, United Nations University, Boschstraat 24, 6211 AX, Maastricht, Netherlands; International Training and Education Center for Health, 325 9th Avenue, Seattle, Washington 98104, USA; Department of Global Health, University of Washington, 3980 15th Ave NE, Seattle, Washington 98105, USA

**Keywords:** two-way texting, ART retention, mHealth, cost effectiveness, HIV care, digital health intervention

## Abstract

Retention in HIV care is crucial for improved health outcomes. Malawi has a high HIV prevalence and struggles with retention despite significant progress in controlling the epidemic. Mobile health (mHealth) interventions, such as two-way texting (2wT), have shown promise in improving antiretroviral therapy (ART) retention. We explore the cost-effectiveness of a 2wT intervention in Lighthouse Trust’s Martin Preuss Center (MPC) in Lilongwe, Malawi, that sends automated SMS visit reminders, weekly motivational messages, and supports direct communication between clients and healthcare workers. Costs and retention (in care at 12 months) rates were compared between 468 2wT and 468 standard of care (SOC) clients. Incremental cost-effectiveness ratios were calculated. Scenario analyses were conducted to estimate costs if 2wT expanded. The 2wT group had higher retention (79%) than SOC (67%) at 12 months post-ART initiation. For 468 clients, the annual costs for 2wT were $36 670.38 compared to SOC’s $33 458.72, with an ICER of $24 705 per additional percent of clients retained. With small populations, 2wT is costlier but more effective. However, expanding 2wT to all new ART clients at MPC would save $105 315 per additional percent of clients retained at 12 months. Scaling-up 2wT to four other high-burden facilities (2901 clients) could save $723 739 per additional percent of clients retained in care, suggesting significant potential cost savings. 2wT appears cost-effective to improve 12-month retention among new ART initiates in this setting. Despite potential limitations, mHealth interventions improve client outcomes and save costs, supporting their integration into HIV care programs.

**RESUMEN:**

La retención de pacientes dentro del sistema de salud es crucial en la atención del VIH para obtener mejores resultados de salud. Malaui tiene una alta tasa de prevalencia de VIH y tiene problemas con la retención, a pesar de haber progresado de manera significativa en el control de la epidemia. Intervenciones de salud móvil (mSalud), como los sistemas de mensajes de texto bidireccionales o de doble vía (2wT), han mostrado promesa en términos de aumentar retención en terapia antirretroviral (TAR). Aquí exploramos la relación costo-efectividad de una intervención 2wT en el centro médico Lighthouse Trust’s Martin Preuss Center (MPC), en Lilongwe, Malaui, que manda con mensajes SMS recordatorios automatizados de visita, frases motivadoras semanales, y apoya la comunicación directa entre clientes y prestadores de salud. Se compararon los costos y las tasas de retención (aún bajo cuidado tras 12 meses) entre 468 clientes con la intervención 2wT y 468 clientes con el estándar de atención. Se calcularon las relaciones de costo-efectividad incremental (RCEI). Se condujeron análisis de escenarios para estimar el costo de expandir la intervención 2wT. El grupo con 2wT presentó mayor retención (79%) que el de atención estándar (67%), a 12 meses de haber iniciado la TAR. Los costos anuales para 468 clientes con 2wT fueron de $36 670.38, contra $33 458.72 de aquellos que recibieron el estándar, con una RCEI de $24 705 por cada percentil adicional de clientes retenidos. Con poblaciones pequeñas, 2wT es más costoso, aunque más efectivo. Sin embargo, si se expandiera el acceso a 2wT a todos los nuevos clientes de TAR en el MPC, se ahorrarían $105 315 por cada percentil adicional de clientes retenidos 12 meses. Ampliar 2wT a cubrir otros cuatro centros con carga elevada de clientes (2901 clientes) podría ahorrar $723 739 por cada percentil adicional de clientes retenidos bajo cuidado, lo cual sugiere un ahorro potencial muy significativo. en este escenario, el uso de 2wT muestra ser rentable y económicamente eficiente en el aumento de la retención por 12 meses de clientes recién iniciados a la TAR. A pesar de presentar algunas limitaciones potenciales, las intervenciones de mSalud mejoran los resultados de salud de los clientes y ahorran costos, apoyando su expedita integración a los programas de cuidado de VIH.

**RESUMO:**

A retenção nos cuidados de saúde para o VIH é crucial para melhorar os resultados em termos de saúde. O Malawi tem uma elevada prevalência de VIH e debate-se com a retenção, apesar dos progressos significativos no controlo da epidemia. As intervenções de saúde móvel (mHealth), como as mensagens de texto bidireccionais (2wT), mostraram-se promissoras na melhoria da retenção da terapia antirretroviral (ART). Exploramos a relação custo-eficácia de uma intervenção 2wT no Centro Martin Preuss (MPC) da Lighthouse Trust em Lilongwe, Malawi, que envia lembretes automáticos de visitas por SMS, mensagens motivacionais semanais, e apoia a comunicação direta entre clientes e profissionais de saúde. Os custos e as taxas de retenção (nos cuidados de saúde aos 12 meses) foram comparados entre 468 clientes de 2wT e 468 clientes de cuidados padrão (SOC). Foram calculados os rácios de custo-eficácia incrementais (ICER). Foram efetuadas análises de cenários para estimar os custos em caso de expansão do 2wT. O grupo 2wT registou uma maior retenção (79%) do que o grupo SOC (67%) aos 12 meses após o início da TAR. Para 468 clientes, os custos anuais do 2wT foram de 36.670,38 dólares em comparação com os 33.458,72 dólares do SOC, com um ICER de 24.705 dólares por percentagem adicional de clientes retidos. Com populações pequenas, o 2wT é mais caro, mas mais eficaz. No entanto, a expansão do 2wT a todos os novos utentes do TARV no MPC pouparia 105.315 dólares por cada percentagem adicional de utentes retidos aos 12 meses. A expansão do 2wT para quatro outras instalações de alta carga (2.901 clientes) poderia economizar US$ 723.739 por percentagem adicional de clientes retidos nos cuidados, sugerindo um potencial significativo de economia de custos. O 2wT parece ser eficaz em termos de custos para melhorar a retenção de 12 meses entre os novos iniciados no TARV neste contexto. Apesar das potenciais limitações, as intervenções de saúde móvel melhoram os resultados dos clientes e poupam custos, apoiando a sua integração nos programas de cuidados do VIH.

**RÉSUMÉ:**

La rétention dans les soins du VIH est cruciale pour améliorer les résultats en matière de santé. Le Malawi a une prévalence élevée du VIH et a des difficultés pour la rétention malgré des progrès significatifs dans le contrôle de l’épidémie. Les interventions de santé mobile (mHealth), telles que les SMS bidirectionnels (2wT), se sont révélées prometteuses pour améliorer la rétention du traitement antirétroviral (ART). Nous explorons le coût-efficacité d’une intervention 2wT au Martin Preuss Center (MPC) du Lighthouse Trust à Lilongwe, Malawi, qui envoie des rappels de visite par SMS automatisés, des messages de motivation hebdomadaires et assiste la communication directe entre les clients et les agents de santé. Les coûts et les taux de rétention (en soins à 12 mois) ont été comparés entre 468 clients 2wT et 468 clients en soins standards (SS). Des rapports coût-efficacité différentiels (RCED) ont été calculés. Des analyses de scénarios ont été menées pour estimer les coûts si l’intervention 2wT s’étendait. Le groupe 2wT présentait une rétention plus élevée (79%) que le groupe SS (67%) 12 mois après le début de l’ART. Pour 468 clients, les coûts annuels du 2wT étaient de 36 670,38 $, contre 33 458,72 $ pour SS, avec un RCED de 24 705 $ par pourcentage supplémentaire de clients retenus. Avec de petites populations, le 2wT est plus coûteux mais plus efficace. Cependant, l’extension du 2wT à tous les nouveaux clients ART du MPC permettrait d’économiser 105 315 $ par pourcentage supplémentaire de clients retenus à 12 mois. L’extension du 2wT à quatre autres établissements à forte charge de travail (2901 clients) pourrait permettre d’économiser 723 739 $ par pourcentage supplémentaire de clients retenus dans les soins, ce qui suggère des économies potentielles importantes. Le 2wT semble rentable pour améliorer la rétention à 12 mois parmi les nouveaux initiés à l’ART dans ce contexte. Malgré leurs limites potentielles, les interventions mHealth améliorent les résultats pour les clients et permettent de réduire les coûts, favorisant ainsi leur intégration dans les programmes de soins du VIH.

## INTRODUCTION

Retention on antiretroviral therapy (ART) among people living with HIV leads to lower mortality and a higher likelihood of viral load (VL) suppression, thereby reducing the risk of HIV transmission [1, 2]. However, ART retention continues to be a major challenge [3, 4], especially within the first 12 months of ART initiation as research, including studies conducted in Malawi has shown that the chances of attrition are highest within the first year of initiating ART [5–9]. Malawi has one of the highest HIV prevalence in the world, with ~9% of the general population living with HIV [10]. Malawi adopted a public health approach with the aim to achieve UNAIDS 95–95-95 targets by 2030 [11]. Progress is commendable: by 2021, 88% of those living with HIV knew their status, 98% of those were on ART, and 97% had their VL suppressed [10]. Despite this significant achievement, Malawi continues to struggle with retention and adherence to ART [12, 13].

Generally, retention in ART care is influenced by several factors, including predisposing factors (e.g. mental illness, substance abuse), client enabling factors (e.g. reminder strategies, transportation) and healthcare environment factors (e.g. provider characteristics, clinic experience [14]. To address these multi-level retention challenges, no single intervention will suffice. However, to contribute to improving ART retention outcomes, the World Health Organization (WHO) recommends evidence-based interventions, including mobile health (mHealth) approaches like reminders that show promise in low-resource settings [15].

Increased access to mobile phones and their rapid technological advancements have led to the development of mHealth as a complementary strategy to strengthen health systems [15]. mHealth is a medical and public health practice that is supported by mobile devices, client monitoring devices, personal digital assistants and other wireless devices [15]. mHealth is a growing strategy with over 600 pilots and programs implemented globally over the last decade [16]. Specific mHealth innovations have shown promise in increasing ART retention in research settings [17].

The use of text messaging, like short messaging service (SMS), for clients and their health providers to communicate has been steadily increasing due convenience, accessibility and privacy advantages [17, 18]. Studies on SMS communication have found that interactive two-way SMS (sending messages with response options) between clients and health providers is more efficacious than one-way informational messaging (sending messages that do not require a response) because it facilitates interaction between the provider and the client [19–21]. SMS can be used to promote adherence by sending prompts to take HIV medication, appointment reminders and interacting with healthcare providers, with results from Kenya, Burkina Faso and Nigeria showing improved uptake and adherence [17, 19, 22, 23]. Furthermore, a meta-analysis by Wald et al. found that two-way messaging was associated with substantially improved medication adherence, compared to one-way text messaging [20]. Based on such evidence, the WHO recommended SMS messaging as an intervention to promote ART [24]. Previous research suggests that two-way SMS interventions could be cost-effective [25, 26], but evidence gaps remain especially in routine, low- and middle-income country (LMIC) settings. As mHealth interventions continue to grow exponentially, there is a critical gap in evaluation and evidence generation to scale only cost-effective, impactful innovations.

Lighthouse Trust (LH) is a WHO-recognized center of excellence for HIV care that has been operational since 2001 [27–29]. LH operates five clinics in Malawi: two in the central district Lilongwe (LH at Kamuzu Central Hospital, Martin Preuss Center (MPC) at Bwaila Hospital), two in the southern districts Blantyre (Umodzi Family Center at Queen Elizabeth Central Hospital) and Zomba (Tisungane Clinic at Zomba Central Hospital) and one in the northern district Mzimba (Rainbow Clinic at Mzuzu Central Hospital) [30].

LH’s MPC clinic in Lilongwe, Malawi has the largest ART cohort in the country, with over 25 000 clients alive in care [31]. At MPC, there are over 7800 monthly visits to the clinic. Approximately 10% of clients at MPC miss their appointments each month. MPC has a policy of following up on those who miss their appointment, which means, on average, 780 clients need tracing monthly. Tracing is done by field tracers who trace clients telephonically or physically to their homes. With such a high demand for client tracing, coupled with limited resources, only about one-third of eligible clients are traced. Lack of, or delays in, tracing reduces ART retention and, ultimately, viral suppression. Poor data quality also hampers tracing effectiveness. On average, of all clients traced, ~55% were not loss-to-follow-up (LTFU) but had transferred to another clinic or were actually still in care, resulting in the inefficient allocation of limited resources [13].

To address this problem, LH collaborated with the International Training and Education Center for Health at the University of Washington, along with technology partner, Medic, and leveraged the open-source Community Health Toolkit (CHT) [32] to customize a two-way texting (2wT) system to enhance early retention among new ART initiates. The 2wT system sends automated visit reminders and weekly motivational messages to clients. Clients can respond to inquiries and send messages to the HCW to change visit dates, report a transfer, or ask for visit-related help. 2wT is free for clients. By directly, and proactively, engaging with clients before a visit is missed, 2wT aimed to improve client outcomes, reducing true LTFU. By identifying transfers and delays before a visit was missed, 2wT aimed to reduce wasted tracing efforts. Results from a quasi-experimental 2wT study at LH’s MPC clinic show that the 2wT system improved 12-month retention in care among new ART initiates by 15%, lowering the risk of LTFU by 66% compared to the standard of care (SOC) approach [33]. Despite initial effectiveness evidence, the cost and cost-effectiveness of this mHealth intervention remain unknown.

Therefore, the primary objective of this study is to assess the cost of implementing the open-source 2wT mHealth intervention and the cost-effectiveness of the intervention in comparison with the SOC buddy approach (visit reminder calls from an expert ART *buddy)* at MPC ART clinic in Lilongwe. In response to recent calls for evidence on SMS innovations and cost-effective interventions from the Malawi Ministry of Health (MOH) [12], this cost and cost-effectiveness analysis from the program perspective may inform feasibility of 2wT scale-up to other MoH facilities.

## MATERIALS AND METHODS

### Study design

This study was based in the LH MPC ART clinic in Lilongwe, Malawi. Using a program (payer) perspective, we conducted a cost-effectiveness analysis comparing 2wT intervention to the SOC. Specifically, we evaluated the costs incurred by the clinic implementing and providing routine retention services (SOC) and the effect of the intervention (2wT) in improving client retention.

**Figure 1:**
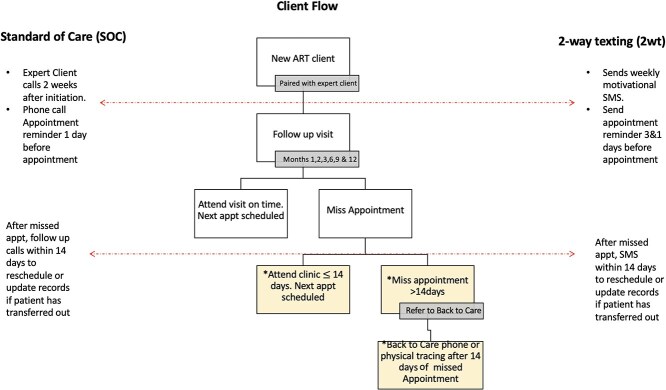
Early retention procedures for SOC and 2wT. Tracing procedures are identical for all clients after a missed appointment of 14 days, regardless of the retention intervention and initiation date

Previously, we conducted a quasi-experimental study to evaluate the impact of 2wT on ART retention over 12 months [33]. The study compared the retention rates of 2wT participants with a control group that received SOC at MPC 1 year before the implementation of 2wT. The intervention group was randomly selected from a pool of 1455 ART clients from the MPC dataset, and participants were matched 1:1 based on age (in 5-year intervals), gender, and WHO stage at treatment initiation. SOC clients were supported with *Buddy* reminder calls before a visit and after a missed appointment, if applicable. 2wT clients were adult clients ages 18 years and older with cell phones who opted into enrollment in the 2wT intervention from June 2021 to April 2022. We calculated the costs of early retention in 2020–2021. All clients were followed for 12 months post-ART initiation, allowing three additional months to complete outcomes. The 2wT and SOC early retention support procedures are presented in Fig. 1. The economic evaluation was conducted following the Consolidated Health Economic Evaluation Reporting Standards 2022 (CHEERS 2022) [34], Appendix 2.

### Standard of care

For client retention, Lighthouse implements a resource-intensive client retention program that is split between early retention for new ART initiates (within the first 12 months of ART initiation) and retention of long-term clients (those on ART longer than 1 year) called Back to Care (B2C). In the early retention program (see Fig. 1), a newly initiated ART client should have ART clinic visits at months 1, 2, 3, 6, 9 and 12 post-initiation for close observation. In addition, most new ART clients are paired with an expert client buddy (EC), who are peer counsellors living with HIV. This pairing is done for the first 12 months of ART initiation. The EC, in addition to providing peer counselling, calls the client for appointment reminders before their scheduled appointment and traces clients through phone calls within 14 days after a missed appointment. If phone tracing is unsuccessful within 14 days and the client has not attended the clinic, the case is sent to B2C for additional tracing, which includes phone calls or home visits. The early retention register documents all phone call reminders and tracing attempts. However, human resource challenges and documentation gaps resulted in only 6 months of recorded buddy data per client.

### The 2wT intervention

Previously described elsewhere [35], the 2wT system replaced phone calls made by ECs for counseling, appointment reminders and missed visit reminders. Clients who fulfilled the eligibility requirements were offered the opportunity to provide their consent and opt-in the 2wT study if they: (i) had initiated ART within the past 6 months, (ii) were aged 18 years or older, (iii) possessed a phone at the time of enrollment, (iv) expressed willingness to receive and send SMS messages, (v) had literacy skills, and (vi) acknowledged and verified their enrollment phone number by receiving and confirming the 2wT enrollment SMS. Clients who either did not have cell phones or chose not to participate were excluded from the study and instead received SOC retention support. The 2wT uses a hybrid workflow that combines: (i) automated workflows that send weekly motivational messages to promote adherence; (ii) individually-tailored SMS reminders to clients with upcoming visits, with a response requested; and (iii) open-ended SMS texts between clients and HCWs that allows clients to reschedule visits, report transfers or report other clinical or non-clinical interactions such as ART side effects or ask questions on their ART care. Appointment reminders are sent 3 and 1 days before the appointment by automated SMS. After a missed appointment, automated SMS reminders are sent within 14 days to reschedule the appointment or update records if the client has transferred to another clinic. If a client enrolled in 2wT has not attended the clinic within 14 days after a missed appointment, the client is referred to B2C for in-person tracing as with SOC. Participants in 2wT did not receive an EC buddy. One 2wT officer is stationed at MPC and handles all client interaction in the system.

### Ethics

For the underlying 2wT implementation effectiveness study, 2wT clients provided written informed consent at opt-in enrollment in either Chichewa or English according to participant preference. The study protocol was approved by the Malawi National Health Sciences Research Committee (#20/06/2565) and the University of Washington, Seattle, USA ethics review board (STUDY000101060). No identifiable data was used in this costing study.

### Data collection

We conducted a time-motion study to record staff time spent on SOC retention and 2wT activities. The data collection tools were developed using Microsoft Excel, provided in Appendix 1. The time and motion study consisted of five days of direct observation at MPC.

#### Outcomes (effects)

The main clinical outcome evaluated was increased retention as defined by those alive and in care at 12 months post ART initiation. Client ART outcome data from both 2wT and comparison clients were extracted from the EMRs, EC tracing registers, and the 2wT database. ART outcome data came from the EMRs, and the SMS data from the 2wT database. The tracing attempts by ECs were documented in the early retention register. We hypothesized that 2wT is more cost-effective for a high-burden ART facility such as MPC.

#### Costs

We used a micro-costing approach for cost estimation. Costing information was obtained from LH expenditure records, payroll information and procurement records and the time motion surveys designed for the study. We identified and categorized all activities and resources involved in both the SOC and 2wT intervention for early retention. These costs were categorized into personnel, training, building utilities, supplies, equipment and communication materials as described in Table 1. The costs were further divided into fixed costs and variable costs, including those that were study-specific and those expected to persist beyond the study period. The fixed costs were one-time expenses, which included 2wT and SOC training and equipment costs for routine retention and study-specific activities. The fixed costs were allocated to SOC and 2wT both interventions based on their proportional utilization of shared resources. The variable costs included recurring costs required to sustain both interventions. These included personnel, communication materials, supplies and building utilities.

**Table 1:** Cost categories

**Cost Category**	**Description**
**Personnel**	Value of personnel time and effort spent in each activity
**Equipment**	Investments that last longer than 1 year, including mobile phones, laptops and furniture
**General supplies**	Supplies used for documentation and contacting clients
**Training**	Expenses used in training sessions for 2wT use and SOC
**Communication**	Costs for phone service companies
**Utilities**	Costs for utilities including electricity, water and internet

Note: SOC, standard of care; 2wT, two-way texting

We quantified and valued the resources for each cost category using Lighthouse expenditure data on salaries and commodity prices. Since the perspective of the analysis was LH (payer) perspective, we excluded costs that are not incurred by the clinic, such as medication costs, which are paid by the government, and study-specific personnel that would not be transferable to routine program implementation.

### Discounting

We used 5% social discount rate as recommended for LMICs due to higher rates of economic growth [36].

### Currency, price date and conversion

All costs were converted from Malawi Kwacha (MWK) to US dollar. We used the 2020–2021 exchange rate of 825 MWK to match the study period of the client outcomes.

### Assumptions

*We had the following assumptions.*

- We assumed the program all staff were working 40 h/week.
- The annual costs for SOC are for all 2678 clients seen in 2020 and could not be easily extracted for 468 clients, only. Therefore, to estimate costs for the 468 clients in SOC, we estimated all personnel and communication costs needed for each client, multiplying that by 468.

### Data analysis

We analyzed the 12 -month ART outcomes for each client and estimated the costs per client enrolled in SOC and 2wT and retained alive and in care at 12-months. We calculated the incremental cost effectiveness ratios (ICERs) of 2wT compared to the SOC to retain clients alive and in care at 12 months [37].

### Sensitivity and scenario analyses

Sensitivity analyses were conducted to investigate how the ICER of the SOC and 2wT changed if 2wT was scaled beyond the pilot, making it accessible to all new ART clients at MPC, and if it was expanded beyond MPC to other facilities. We calculated the ICERs for both scenarios.

Scenario 1 (scale up to all new ART initiates at MPC): We assumed that 44% of all new initiates at MPC in 2020 would be eligible for 2wT (according to enrollment screening data). Therefore, of the 2678 initiated on ART at MPC in 2021, we estimated the cost of providing retention support to 1005 in SOC and 942 clients in 2wT. In the SOC, the personnel and communication costs were estimated by multiplying the unit cost per client of the base case by the number of clients enrolled. The supplies, utilities, training and equipment would remain the same. It is estimated that one full-time FTE 2wT data officer can manage up to 3000 clients.

Scenario 2 (scale up to four additional facilities): The second scenario estimated costs if 2wT was scaled to the other four LH high-burden ART facilities across the country which enrolled 2901 new ART clients in 2022. It is estimated that the existing 2wT data officer and a retention assistant would be enough to manage the 2wT system and client enrolment in this scenario. The enrollment eligibility was presumed the same as from screening data. For personnel, communication and supplies cost, we multiplied the unit cost per client from the base case by the number of clients. We assumed the utilities, training, and equipment costs would be the same for each facility. For 2wT, one 2wT-specific data officer from MPC would serve multiple sites, but each facility would use an EC at 20% FTE for enrolling new clients only. For communication and supplies costs, we multiplied the unit cost per client from the base case by the number of clients. Each facility would have the same equipment costs, excluding a lockable cabinet and 2wT system maintenance costs since the central 2wT system would be managed and maintained at MPC. For training costs, the ECs would only need 2wT-specific training, but not retention program training, since they would have already received that as part of their normal duties.

## RESULTS

### Demographics and retention outcomes

Table 2 presents the baseline characteristics and 12-month ART outcomes of clients included in SOC and 2wT. Retrospectively, 468 adult new ART clients aged 18 and over with a registered mobile phone number were randomly selected in SOC for comparison with the number of 468 participants who were enrolled in the 2wT intervention. As shown in Table 2, in both SOC and 2wT intervention groups, there were more women than men (56%) and the median age was 33 years, with more participants in the 25–44 age group. The majority (78%) of the participants were initiated on ART at WHO stage 1 or 2, reflecting the demographic profile of new ART clients at MPC [31]. At 12 months post-ART initiation, 2wT intervention had more (79%) clients retained alive in care compared to those in the SOC (67%) (*P* < 0.001). In addition, SOC had more clients LTFU (18% vs 6%) (*P* < 0.001), and a higher rate of clients who stopped ART (6% vs 2%) (*P* = 0.004) compared to the 2wT. The transfer-out rate and death rate were the same for both interventions at 9% and 1%, respectively.

**Table 2:** Baseline characteristics and 12-month ART outcomes of clients included in SOC and 2wT at Martin Preuss Centre, Lilongwe, Malawi

**Characteristic**	**SOC, N = 468**		**2wT arm, N = 468**		***P*-value**
**Sex**					
Male	261	56%	261	56%	
Female	207	44%	207	44%	
**Age Median, (IQR)**	33 (27,40)		33 (27, 40)		
**Age group**					
18–24	68	15%	68	15%	
25–34	190	41%	190	41%	
35–44	146	31%	147	31%	
45+	64	14%	63	13%	
**WHO stage (severity of HIV disease)**					
1 or 2	364	78%	364	78%	
3	71	15%	71	15%	
4	30	6%	30	6%	
Unknown	3	1%	3	1%	
**ART outcomes**					
Alive in care	372	79%	312	67%	<0.001
LTFU	26	6%	82	18%	<0.001
Stopped treatment	10	2%	27	6%	0.004
Transfer out	37	8%	40	9%	0.721
Dead	7	1%	7	1%	1
Discontinue SMS/Withdraw	16	3%	0	0%	<0.001

Note: SOC, standard of care; 2wT, two-way texting; IQR, inter-quartile range; WHO, world health organization; LTFU, loss to follow up; ART, anti-retroviral therapy; SMS, short messaging service.

### Costs

Table 3 summarizes the total annual costs and unit costs for implementing SOC services and the 2wT intervention. The annual costs for 2wT were higher at $36 670.38 compared to $33 458.72 for SOC. Both fixed costs and variable costs were greater for 2wT. Personnel costs constituted the highest expense for 2wT, while the building costs were the highest expense for SOC. Overall, the cost per client enrolled was higher for 2wT at $78.36 compared to $71.49 for SOC, with a difference of $6.87 between the two. However, the cost per client retained in care after 12 months was higher for SOC ($107.24 vs $98.58) due to more clients retained in care for 2wT.

**Table 3:** Total annual costs and unit costs of SOC and 2wT

	**Total cost**		**Cost per client**		**Cost per client retained** **at 12mos**	
**Categories**	**SOC**	**2wT**	**SOC, N = 468**	**2wT, 468**	**SOC, N = 312**	**2wt, N = 372**
**Fixed Costs (one-time)**						
**Training**	$6592.18	$9285.32	$14.09	$19.84	$21.13	$24.96
**Equipment**	$3603.03	$8909.44	$7.70	$19.04	$11.55	$23.95
**Variable Costs (recurrent)**						
**Personnel**	$9597.10	$15 187.17	$20.51	$32.45	$30.76	$40.83
**Supplies**	$4743.15	$111.15	$10.13	$0.24	$15.20	$0.30
**Communication**	$87.27	$1395.37	$0.19	$2.98	$0.28	$3.75
**Utilities**	$8835.99	$1781.92	$18.88	$3.81	$28.32	$4.79
**Total**	**$33 458.72**	**$36 670.38**	**$71.49**	**$78.36**	**$107.24**	**$98.58**

Note: SOC, standard of care; 2wT, two-way texting.

We also assessed the costs per gender, age group and WHO stage. Notably, in general, the costs were higher for 2wT in all categories except for the unknown WHO stage.

The cost shares by input categories for all costs are shown in Appendix 3. The personnel, training, equipment, and communication costs were higher for 2wT at 41%, 25%, 24% and 4%, respectively, compared to the respective allocations of 29%, 20%, 11% and 0.3% for SOC. Conversely, the utilities and general supplies were higher for SOC services, with 26%, and 14%, respectively, in contrast to 5% and 0.3% for 2wT.

### Scenario analysis

Tables 4 and 5 present the annual and unit costs for SOC and 2wT for scenarios 1 and 2, respectively.

**Table 4:** Scenario 1—total and unit costs of SOC and 2wT

**Categories**	**Total cost**		**Cost per client**		**Cost per client retained at 12 months**	
	**SOC**	**2wT**	**SOC, N = 1500**	**2wT, 1178**	**SOC, N = 1005**	**2wt, N = 931**
**Fixed Costs (one-time)**						
**Training**	$6592.18	$9285.32	$4.39	$7.88	$6.56	$9.97
**Equipment**	$3603.03	$8909.44	$2.40	$7.56	$3.59	$9.57
**Variable Costs (recurrent)**						
**Personnel**	$30 753.37	$19 633.22	$20.50	$16.67	$30.61	$21.09
**Supplies**	$4743.15	$111.15	$3.16	$0.09	$4.72	$0.12
**Communication**	$279.66	$1395.37	$0.19	$1.18	$0.28	$1.50
**Utilities**	$8835.99	$1781.92	$5.89	$1.51	$8.79	$1.91
**Total**	**$54 807.37**	**$41 116.44**	**$36.54**	**$34.90**	**$54.55**	**$44.17**

Note: SOC, standard of care; 2wT, two-way texting.

**Table 5:** Scenario 2—total and unit financial costs of SOC and 2wT

**Categories**	**Total cost**		**Cost per client**		**Cost per client retained at 12 months**	
	**SOC**	**2wT**	**SOC, N = 1625**	**2wT, 1276**	**SOC, N = 1088**	**2wt, N = 1008**
**Fixed Costs (one-time)**						
**Training**	$26 368.74	$10 772.56	$16.23	$8.44	$24.23	$10.68
**Equipment**	$14 412.10	$3802.60	$8.87	$2.98	$13.24	$3.77
**Variable Costs (recurrent)**						
**Personnel**	$33 314.23	$13 436.61	$20.50	$10.53	$30.61	$13.32
**Supplies**	$16 464.82	$303.16	$10.13	$0.24	$15.13	$0.30
**Communication**	$302.95	$3805.79	$0.19	$2.98	$0.28	$3.77
**Utilities**	$35 343.94	$0.00	$21.75	$0.00	$32.47	$0.00
**Total**	**$126 206.78**	**$32 120.72**	**$77.67**	**$25.17**	**$115.95**	**$31.85**

Note: SOC, standard of care; 2wT, two-way texting.

As shown in Table 3, expanding 2wT to all new ART clients at MPC (scenario 1) with the assumption that 44% would enroll in 2wT, would decrease the total costs by 63.8% and 12.1% for SOC and 2wT, respectively. The increase in SOC cost was driven primarily by personnel costs, as more EC personnel would be needed for more clients. For 2wT, there was only a minor increase in the 2wT data officer FTE, increasing to 100%. Expanding to four other LH clinics (scenario 2), as shown in Table 5, would increase the cost per client by 277.2% for SOC, but decrease by 12.4% for 2wT. The main cost driver for SOC was personnel, utilities costs and fixed costs. The 2wT costs slightly decreased due to reduced personnel costs and no utilities costs. In scenario 2, with 2wT scaling up, 2wT the unit cost decreases.

### Incremental ICER

Table 6 describes the ICER in the scenario analyses. In the base case scenario with 468 participants in each group, SOC costs $33 458.72 with a unit cost of $107.58 and 2wT costs $36 670.38 with a unit cost of $98.05. The ICER for 2wT compared to the SOC was +$26 763.86 per additional percent of clients retained alive and in care: 2wT is more costly, but also more effective, compared to the SOC to retain clients in care.

**Table 6:** Change in cost and ICER with different 2wT scale-up scenarios

**Scenarios**	**SOC**		**2wT**		**ICER**
	Total cost	Unit cost at 12 months	Total cost	Unit cost at 12 months	
Base scenario (N = 468 for both SOC and 2wT)	$33 458.72	$107.58	$36 670.38	$98.05	+$26 763.86
Scenario 1: If all new ART clients had access to 2wT (N = 2678) at MPC	$54 807.37	$54.53	$41 116.44	$43.65	-$114 091.14
Scenario 2: If 2wT is scaled to the other 4 LH clinics (N = 2901)	$126 206.78	$112.58	$32 120.72	$31.46	-$784 051.51

Note: SOC, standard of care; 2wT, two-way texting; ART, antiretroviral therapy; MPC, Martin Preuss Center; LH, Lighthouse Trust.

In the first scenario where 2wT is extended to all new ART clients at MPC (N = 2678), the unit cost drops significantly to $54.53 (total cost $54 807.37) for SOC, and $43.65 (total cost of $41 116.44) for 2wT. This shift results in a substantial decrease in ICER for 2wT, transitioning from +$26 763.86 to -$114 091.14 (500% lower), per additional percent of clients retained alive and in care. In this scenario, 2wT is both more effective and less costly than SOC. Finally, in the second scenario where 2wT is implemented at four other LH clinics (N = 2901), 2wT becomes even more cost-effective with a unit and total cost of $31.46 and $32 120.72, respectively, and an ICER of -$784 050.51 per additional percent of clients retained in care. This indicates substantial cost savings for 2wT compared to SOC at scale.

## DISCUSSION

We conducted a comprehensive cost-effectiveness analysis based on a payer costs (program/organization level) to evaluate the impact of a 2wT mHealth intervention on ART retention among newly initiated clients in a public ART clinic in Lilongwe, Malawi, comparing 2wT with the SOC. Personnel costs constituted the largest expense for 2wT, while utilities costs were the highest for SOC. The total annual costs for 2wT were slightly higher than SOC costs; however, higher 2wT client retention resulted in a lower cost per client retained in care after 12 months as compared to SOC. Our analysis also underscores the large potential cost-effectiveness of the 2wT intervention when implemented at scale. In consideration of our previous results indicating that 2wT clients were more likely to be retained at 12 months post ART initiation (92%) as compared to their SOC peers (76%), investment in 2wT appears advisable in routine LMIC ART settings. We discuss several areas of interest for consideration of 2wT expansion.

First, although 2wT does not appear cost-effective with the small client enrollment numbers of the study context, 2wT may bring large gains if implemented at scale. We explored two scenarios for our sensitivity analyses: one examining the expansion of 2wT to all new ART clients and another considering its implementation across LH facilities. Our study results suggest that, as 2wT expands, its unit costs would decrease. Furthermore, the 2wT intervention resulted in an ICER of $24 705 per additional percentage of retention. Expanding 2wT for all new ART initiates at MPC would significantly reduce the unit costs. Likewise, further scale-up of 2wT to four additional clinics would reduce costs dramatically. The cost drivers in both scenarios are primarily personnel and utilities. The higher personnel costs in SOC stem from the increased need for human resources, like ECs, to manage the larger number of clients. In contrast, the 2wT intervention relies more on technology, which requires fewer additional personnel to scale up, thus resulting in lower relative personnel costs despite the increase in clients. Additionally, SOC requires an entire building to support the team needed to provide comprehensive care for ART clients, whereas the 2wT system only needs to be hosted at one computer at the facility, using a hub-to-spoke model to reach clients from other clinics. This hub-and-spoke 2wT expansion approach would incur minimal, additional on-site costs to support existing personnel who would only need to work part-time to enroll clients from other facilities into the central 2wT system. Overall, in either scenario, the ICER had negative values, indicating improved outcomes and cost savings. These findings collectively emphasize the potential advantages of the 2wT intervention across diverse scenarios, showcasing improved client retention and suggesting the feasibility of achieving cost savings through strategic implementation.

Second, our study findings align with previous cost-effectiveness analyses that suggest SMS interventions become more cost-effective when implemented at scale [26, 38, 39]. However, this recognition comes with an important caveat for future mHealth cost-effectiveness studies. It is difficult to test and evaluate the cost-effectiveness of an mHealth intervention at scale where larger populations absorb mHealth implementation running costs. mHealth interventions should first be tested with small samples during the development and pilot stages, proving through rigorous evaluation that the intervention works. Although this sets a high bar for expansion, generating evidence before expansion avoids wasting resources on interventions that are not effective. In our costing study, we demonstrated the cost-effectiveness of 2wT at 12 months, first, and employed a well-informed scenario analysis to explore the potential added value at scale. We will use this evidence and rigorous data to advocate for scaling 2wT.

Additionally, a systematic review of economic evaluations on mHealth interventions identified SMS interventions based on behavior change theory and fit for context as key drivers for successful mHealth strategies [40]. The findings of this cost-effectiveness analysis support the use of the 2wT mHealth intervention as a cost-effective method for improving ART retention in a large public HIV clinic in Malawi. The 2wT was deliberately built using an open-source CHT app, a global digital good, and as such, there are no licensing fees associated with the app use. The absence of licensing fees contributes to the reduction in the total cost of ownership, a key consideration for MOH decision-making process regarding potential national-scale implementation of the 2wT. Also in previous research, the 2wT intervention has been shown to be effective, usable and acceptable [35] among new ART initiates for improving early retention at MPC. The 2wT format confirms delivery of SMS sent, and it allows the client to respond at their convenience and discreetly, whereas unanswered phone calls may not register as missed calls to the recipient, and there are no voicemail options for Malawi phone plans. The 2wT is also free, thereby encouraging client feedback. Additionally, the 2wT intervention is also beneficial to the healthcare facility by helping HCWs streamline their workload by reducing unnecessary tracing and automating the generation of defaulter lists (lists of those who missed visits by more than 14 days), which can lead to improved efficiency and resource allocation within the healthcare system [41].

Implementing mHealth interventions for ART adherence at scale appears feasible and cost-effective. These interventions can build on existing healthcare infrastructure and leverage available resources [5]. In this study, the 2wT intervention was implemented to improve routine services using routine healthcare worker resources [35]. Given the substantial cohort of nearly 26 000 ART-enrolled clients at MPC, coupled with an annual influx of ~2000 new ART clients, we recommend that 2wT be used to supplement the existing early retention program, emphasizing the importance of ensuring minimal additional workload for HCW for optimal effectiveness. In addition, robust training and mentorship for proper implementation is imperative. It is noteworthy that because HCWs at MPC were involved in the development of the 2wT system, they may have greater buy-in- a dynamic that may differ when scaling the system to other LH facilities when the system is already developed.

Although the 2wT approach to engage clients in care appears robust at both Lighthouse and in other settings, [25, 42] potential challenges to scaling up were acknowledged. Implementing 2wT requires appropriate training, practice-based mentoring and constant supervision. The 2wT retention approach also required generating client demand for 2wT—which may require additional education, outreach and engagement activities not considered in this analysis. Enrolling clients in 2wT beyond their ART initiation visits (for clients who are already in care) would necessitate establishing a supportive national policy for SMS-based retention including, e.g. enabling policy for minors to enroll with guardian support. Lastly, with any new retention support, there are reporting redundancies that should be addressed to enhance efficiency.

Our study provides crucial insights into the cost implications of implementing a mHealth intervention within a routine setting. Notably, the early retention program at MPC is just one piece of a larger effort to keep clients engaged in care. MPC offers a wide range of HIV care and treatment services across the HIV cascade [29, 30]. Because of this setup, at MPC, the HCWs have varied roles and responsibilities that often cut across different departments. As HCWs take on more senior positions, their roles become even more complex and overlapping, adding challenge to accurately calculate the specific costs associated with the early retention services within the regular program. Thus, in addition to the time-motion study, we had to continuously refine our estimates with the HCWs involved in retention services to accurately estimate the different cost categories. MPC has detailed records for documenting clinic visits through the EMRs and individual client files.

### Limitations

It is essential to acknowledge some limitations of this study. Firstly, not all clinic operational costs, such as senior leadership personnel expenses, were incorporated, potentially leading to an underestimation of total costs. The SOC costs were extracted from the overall MPC program expenditure data; the absence of specific SOC intervention-based expenditure recording within LH accounting records could have omitted certain costs. Utility or building costs could vary more than estimated. Furthermore, in 2020, EC buddy records were incomplete. Consequently, the frequency of EC interactions with the clients and referrals to B2C could not be completely ascertained. Moreover, the 2wT is limited to those who have exclusive use of a mobile phone and are literate. This highlights the need to consider 2wT as an augmentation or complement to SOC and not a replacement. 2wT is not yet integrated into the underlying MPC electronic medical record system; however, planned integration should further lower costs through more automated visit reminders and referrals to B2C. Lastly, the underlying quasi-experimental design of the 2wT study, itself, could be strengthened; a randomized control trial of 2wT versus SOC to improve 12-month outcomes is forthcoming. Despite these limitations, the 2wT intervention should still be considered an effective intervention for those who can use it, as it has demonstrated positive outcomes in improving ART retention.

## CONCLUSION

Based on this study, we conclude that 2wT is a cost-effective method to improve early retention of new ART initiates in Lilongwe, Malawi. 2wT reduced cost per retained client when compared to those receiving the SOC. Our scenario analyses demonstrate that as the 2wT is expanded in scale, it may yield cost savings, thereby establishing its cost-effectiveness for the clinic. The 2wT intervention should not replace the existing SOC services, rather, it should complement the existing retention interventions to support engagement in care. This study sets the stage for future investigations aimed at assessing the cost-effectiveness of scaling up the 2wT program, measuring 2wT impact on timely visit attendance, client retention, attrition and re-engagement in care over time. The results of this study will also be shared with the MOH, and other key stakeholders to advocate for the broader adoption and scaling of mHealth interventions within HIV care programs in Malawi and similar high-burden settings. By integrating these interventions, healthcare systems can enhance client retention, optimize resource utilization and improve health outcomes for individuals living with HIV.

## Supplementary Material

Supplementary_information_for_review_oqae030

## Data Availability

Interested researchers may contact the Human Subjects Division representative for our study at jedelson@uw.edu to request access to the datasets. Access to data will be restricted to those who complete data sharing agreements.
