# Supplementary material for: Cost-effectiveness analysis of a two-way texting (2wT) intervention to improve ART retention among newly-initiated antiretroviral therapy clients in Malawi
Source: Oxf Open Digit Health. 2024 Dec 2;2(Suppl 2):ii45–55. doi: 10.1093/oodh/oqae030 (PMC11936329; doi:10.1093/oodh/oqae030)

**Supplementary Information:**

**Cost-effectiveness analysis of a two-way texting (2wT) intervention to improve ART retention among newly-initiated antiretroviral therapy clients in Malawi**

Christine Kiruthu-Kamamia^1,2,3*^, Hiwot Weldemariam^4^, Mirriam Chipanda^2^, Jacqueline Huwa^2^, Johnnie Seyani^2^, Harrison Chirwa^2^, Aubrey Kudzala^2^ , Agnes Thawani^2^, Joseph Chintedza^2^, Odala Sande^2^, Geldert Chiwaya^2^, Hannock Tweya^3,5^, Milena Pavlova^1^, Wim Groot^1^, Caryl Feldacker^3,5^

**Affiliations**

^1^United Nations University – Maastricht Economic and Social Research Institute on Innovation and Technology, Maastricht, Netherlands

^2^Lighthouse Trust, Lilongwe, Malawi

^3^International Training and Education Center for Health, Seattle, Washington, USA

^4^Department of Epidemiology, University of Washington, Seattle, Washington, USA

^5^Department of Global Health, University of Washington, Seattle, Washington, USA

***Corresponding Author:**

Christine Kiruthu-Kamamia, MPH

United Nations University – Maastricht Economic and Social Research Institute on Innovation and Technology

Boschstraat 24, 6211 AX Maastricht, Netherlands

Email: c.kiruthu-kamamia@student.maastrichtuniversity.nl

Phone: 265992464418

## Supplementary Materials

### Appendix 1: Data Collection Tools for time-motion study

**Time and motion observation tool for health care workers**

| Data Collection Information  *PLEASE FILL OUT THIS SECTION AT THE BEGINNING OF THE DAY, BEFORE BEGINNING OBSERVATIONS.* | |
| --- | --- |
| 1. **Date** | Day       Month            Year |
| 1. **Time arrived at facility** | \|__\|__\|😐__\|\|__\| |
| 1. **Data collector initials** | \|__\|__\|__\| |
| 1. **Facility name** | Lighthouse  MPC |
| 1. **Retention group** | 󠄀 2 WT  󠄀 Routine |

| Consent | |
| --- | --- |
| 1. **Did health care worker provide consent to participate?** | 󠄀 Yes. Proceed with data collection.  󠄀 No. STOP. |

*AFTER CONSENT, INTRODUCE SELF TO HEALTH CARE WORKER:* Thank you for agreeing to participate in this full-day observation. My name is ______. Today, I’d like to follow you to observe you providing health services to clients. I am interested in particular in how much time you spend providing care for retention activities as compared to other services. We will document the task you are performing, where you are performing it, and document the start and end time of each task. **We are not assessing your performance**, we just want to better understand the order of the daily work and the amount of time it takes. If you could please do your work today as you usually would, that would be most useful for us. We will not speak and do our best to stay out of your and your clients’ way so as not to be disruptive.

| Health Care Worker Information | |
| --- | --- |
| 1. **Health care worker ID** |  |
| 1. **Provider cadre**   **(SPELL OUT each acronym)** | 󠄀 B2C field tracer  󠄀 B2C phone tracer  󠄀 Retention Assistant (RA)  󠄀 Retention Coordinator  󠄀 Health Promoter (HP)  󠄀 Expert client (EC)  󠄀 Community Care Supporter (CCS)  󠄀 Retention Supervisor  󠄀 Senior Tracer  󠄀 2 WT Officer (data officer)  󠄀 2 WT Officer (RA)  󠄀 Other (please explain): _________________ |
| 1. **Length of time at this facility at your current cadre** | 󠄀 <1 year  󠄀 1-5 years  󠄀 6-10 years  󠄀 More than 10 years |
| 1. **Highest Academic qualification** | 󠄀 Diploma or above  󠄀 Certificate  󠄀 MSCE  󠄀 JCE/PSL  󠄀 Other |
| 1. **Main role(s) TODAY**   ***Check all that apply*** | 󠄀 Remind ART clients about their upcoming clinic appointment  󠄀 Phone tracing clients who miss their clinic appointment/LTFU/VL appointment (tracing and interview)  󠄀 Field tracing clients who miss their clinic appointment/LTFU/VL appointment (tracing and interview)  󠄀 Conduct ART adherence counselling or education session  󠄀 Adherence promotion and interaction by phone (SMS or phone call)  󠄀 Fill and update locator form, update records  󠄀 Compile reports, generate lists of LTFU appointment  󠄀 Supervision  󠄀 Other (please explain): _____________________________________ |

### Appendix 2: Consolidated Health Economic Evaluation Reporting Standards 2022 (CHEERS 2022)

From: [Consolidated Health Economic Evaluation Reporting Standards 2022 (CHEERS 2022) statement: updated reporting guidance for health economic evaluations](https://bmcmedicine.biomedcentral.com/articles/10.1186/s12916-021-02204-0)

| **Section/topic** | **Item No** | **Guidance for reporting** | **Reported in Page** |
| --- | --- | --- | --- |
| **Title** | | | |
| Title | 1 | Identify the study as an economic evaluation and specify the interventions being compared. | 1 |
| **Abstract** | | | |
| Abstract | 2 | Provide a structured summary that highlights context, key methods, results, and alternative analyses. | 2 |
| **Introduction** | | | |
| Background and objectives | 3 | Give the context for the study, the study question, and its practical relevance for decision making in policy or practice. | 3-5 |
| **Methods** | | | |
| Health economic analysis plan | 4 | Indicate whether a health economic analysis plan was developed and where available. | - |
| Study population | 5 | Describe characteristics of the study population (such as age range, demographics, socioeconomic, or clinical characteristics). | 5 |
| Setting and location | 6 | Provide relevant contextual information that may influence findings. | 5 |
| Comparators | 7 | Describe the interventions or strategies being compared and why chosen. | 6 |
| Perspective | 8 | State the perspective(s) adopted by the study and why chosen. | 5 |
| Time horizon | 9 | State the time horizon for the study and why appropriate. | 5 |
| Discount rate | 10 | Report the discount rate(s) and reason chosen. | 7 |
| Selection of outcomes | 11 | Describe what outcomes were used as the measure(s) of benefit(s) and harm(s). | 6 |
| Measurement of outcomes | 12 | Describe how outcomes used to capture benefit(s) and harm(s) were measured. | 6 |
| Valuation of outcomes | 13 | Describe the population and methods used to measure and value outcomes. | 6 |
| Measurement and valuation of resources and costs | 14 | Describe how costs were valued. | 7 |
| Currency, price date, and conversion | 15 | Report the dates of the estimated resource quantities and unit costs, plus the currency and year of conversion. | 7 |
| Rationale and description of model | 16 | If modelling is used, describe in detail and why used. Report if the model is publicly available and where it can be accessed. | - |
| Analytics and assumptions | 17 | Describe any methods for analyzing or statistically transforming data, any extrapolation methods, and approaches for validating any model used. | 7-8 |
| Characterizing heterogeneity | 18 | Describe any methods used for estimating how the results of the study vary for subgroups. | 7-8 |
| Characterizing distributional effects | 19 | Describe how impacts are distributed across different individuals or adjustments made to reflect priority populations. | 7-8 |
| Characterizing uncertainty | 20 | Describe methods to characterize any sources of uncertainty in the analysis. | 7-8 |
| Approach to engagement with clients and others affected by the study | 21 | Describe any approaches to engage clients or service recipients, the general public, communities, or stakeholders (such as clinicians or payers) in the design of the study. | - |
| **Results** | | | |
| Study parameters | 22 | Report all analytic inputs (such as values, ranges, references) including uncertainty or distributional assumptions. | - |
| Summary of main results | 23 | Report the mean values for the main categories of costs and outcomes of interest and summarise them in the most appropriate overall measure. | 8-9 |
| Effect of uncertainty | 24 | Describe how uncertainty about analytic judgments, inputs, or projections affect findings. Report the effect of choice of discount rate and time horizon, if applicable. | 9-10 |
| Effect of engagement with clients and others affected by the study | 25 | Report on any difference client/service recipient, general public, community, or stakeholder involvement made to the approach or findings of the study | - |
| **Discussion** | | | |
| Study findings, limitations, generalizability, and current knowledge | 26 | Report key findings, limitations, ethical or equity considerations not captured, and how these could affect clients, policy, or practice. | 10-13 |
| **Other relevant information** | | | |
| Source of funding | 27 | Describe how the study was funded and any role of the funder in the identification, design, conduct, and reporting of the analysis | - |
| Conflicts of interest | 28 | Report authors conflicts of interest according to journal or International Committee of Medical Journal Editors requirements. | - |

### **Appendix 3: Cost shares by input categories for all costs**


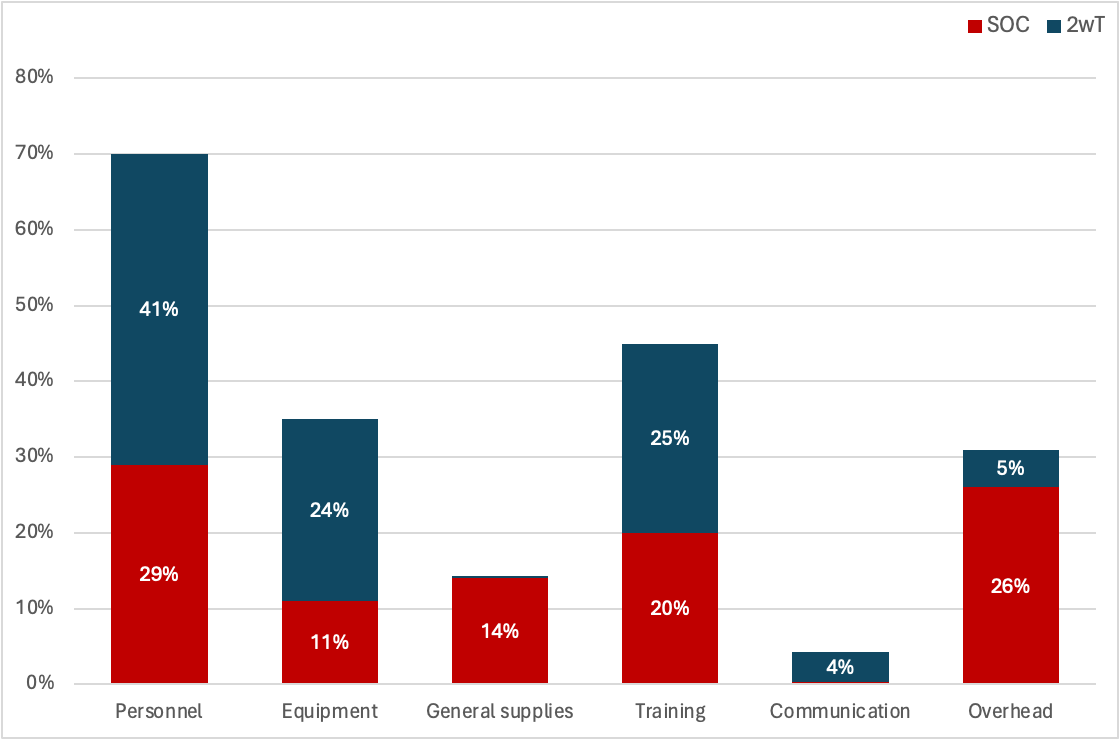

Supplement: Supplementary_information_for_review_oqae030 [file Supplementary_information_for_review_oqae030.docx]
